# Factors affecting oral frailty and nutritional status in older adults living alone

**DOI:** 10.3389/fnut.2025.1586860

**Published:** 2025-06-04

**Authors:** Ji-Min Cha, Dong Hoon Jung, Ha-neul Kim, Hee-Sook Lim

**Affiliations:** Department of Gerontology, AgeTech-Service Convergence Major, Graduate School of East-West Medical Science, Kyung Hee University, Yongin, Republic of Korea

**Keywords:** older adults, single-person households, oral frailty, nutrition, isolation

## Abstract

**Introduction:**

The global aging population is rapidly increasing, with South Korea experiencing the fastest aging rate among the OECD countries. Consequently, the number of older adults living alone is sharply increasing, creating an urgent social issue that necessitates comprehensive and systematic care service policies. Frailty is a key indicator of the overall health status of older adults, and because oral health and nutrition are closely linked, continuous monitoring is essential in this population.

**Methods:**

This study aimed to analyze the factors influencing oral frailty and nutritional status among older adults living alone in South Korea by using an oral frailty screening tool, which is one of the recently developed function-specific frailty screening tools. A survey of 606 adults aged 65 years and older examined their sociodemographic characteristics, health status, oral health, and nutritional status. Logistic regression analysis identified key factors affecting oral frailty and nutritional status.

**Results:**

Results revealed a direct association between oral frailty and nutritional status, with loneliness emerging as a common factor influencing both variables.

**Discussion:**

These findings highlight the importance of maintaining a balanced diet alongside proper oral hygiene and emphasize the need for integrated interventions including emotional support. Therefore, this study underscores the need for continuous care strategies to promote healthy aging and improve the quality of life of older adults.

## 1 Introduction

The global population officially became an aging society in 2002, and by 2023, the number of people aged 65 years and older had reached 810 million (10.0%) worldwide (1). According to the World Health Organization, the global population aged 60 years or older is expected to double from 1 billion in 2020 to 2.1 billion by 2050 (2), with rapid increases projected not only in developed regions such as Europe and North America but also in Asia and Central and South America (3). These global sociodemographic changes underscore the importance of preparing health and social care systems across different regions. In this context, South Korea, with its rapidly aging population and growing number of older adults living alone, offers meaningful insights that may be relevant beyond its national context. These global trends are particularly evident in South Korea, where the aging process is occurring at an unprecedented pace.

In terms of household composition, older adults living alone or as couples accounted for two-thirds of all households that had older adults; and these numbers have been steadily increasing. The proportion of older adults living alone is projected to rise from 28% in 2018 to 35% by 2050 (4). Of the 37 member countries that are part of the Organization for Economic Co-operation and Development (OECD), South Korea has the fastest aging rate with an annual average increase of 4.4%. The proportion of older adults living alone is also rapidly increasing (5), with 1.92 million single-person, older adult households (26.0%) as of 2023. Of these, 19.1% households consisted of adults aged 70 years and older, and this figure is expected to increase 2.6 times by 2052 (6). This growing trend of older adults living alone poses a grave societal concern, necessitating comprehensive national care policies, as without appropriate intervention, long-term social costs will continue to rise (7).

Household type plays a crucial role in determining the quality of life and health status of older adults by distinguishing them from other age groups (8). Compared to older adults living in multi-person households, those living alone are more vulnerable to social isolation, depression, and psychological and financial instability (8). Prolonged solitary living can lead to a decline in physical function, negatively affecting overall health. Additionally, a lack of social interaction can impair brain activity and accelerate cognitive decline (9).

Frailty is a critical indicator of an older adult’s overall health status, representing a condition where multiple functions decline simultaneously (10). Early screening, individualized intervention plans, and appropriate management are essential for addressing frailty (11). Various well-established tools are used to conduct objective frailty screening and assessments, with function-specific screening methods such as oral frailty screening gaining increasing attention (12). Oral health problems such as dental caries, periodontitis, tooth loss, and dry mouth are highly prevalent among older adults. Impaired masticatory function can lead to nutritional imbalances, ultimately increasing immune dysfunction, inflammation, chronic disease severity, and mortality risk (13, 14). In 2023, 50.4% of South Korean older adults aged 70 years and older had fewer than 20 remaining teeth, and 37.9% reported chewing difficulties. However, the rate of regular oral health checkups among older adults remains low at only 30% (15). Additionally, approximately 20% of older adults experience swallowing difficulties, with over 90% of them at risk of malnutrition (16). According to the Health Insurance Review & Assessment Service, the number of medical visits due to swallowing difficulties reached 26,818 in 2022, reflecting a 200% increase over the past decade (15). A four-year observational study in Japan found that older adults with oral frailty were more than twice as likely to develop sarcopenia, disabilities, and increased mortality rates, emphasizing the importance of oral frailty prevention in systemic health management (17).

Given the direct and reciprocal relationship between oral health and nutrition, continuously monitoring their interactions is necessary (18). Studies have found that older adults with chewing difficulties consume significantly fewer vegetables and fruits than those with normal chewing ability, affecting their overall dietary quality (19, 20). Additionally, chewing enhances taste perception, and difficulty chewing can lead to decreased appetite, altered food choices, and nutritional imbalances (21). Compared to older adults living in multi-person households, those living alone face a higher risk of malnutrition because of their insufficient nutrient intake. Their limited dietary choices may contribute to malnutrition, frailty, and sarcopenia, further exacerbating oral frailty (22).

Despite these concerns, there is limited research that holistically evaluates and analyzes the oral health and nutritional status of older adults living alone. This study aims to comprehensively assess the nutritional status, health conditions, and oral health of older adults living alone in South Korea while identifying key factors influencing oral frailty and malnutrition. Through these findings, this study highlights the need for systematic oral care and nutritional support for older adults and provides essential epidemiological data to support health promotion strategies.

## 2 Materials and methods

### 2.1 Study design and participants

Study participants were recruited through local support centers for older adults living alone, with assistance from the Comprehensive Support Center for the Elderly Living Alone. These support centers operate under the Comprehensive Support Center for the Elderly Living Alone, an institution under the Korea Ministry of Health and Welfare. Participants comprised 607 older adults aged 65 years and above who independently managed activities of daily living, such as cooking and sleeping. After excluding one individual who withdrew, the final dataset comprised 606 participants. Participants were classified into three groups (normal, at-risk, and high-risk) based on their oral frailty risk, assessed using the Korean Oral Frailty Risk Screening Questionnaire (12). The oral frailty risk assessment included 12 items related to chewing difficulty, dry mouth, pronunciation difficulty, oral hygiene practices, and overall oral satisfaction. The maximum total score achievable was 19.5, and participants were categorized as normal (0–0.5), at-risk (1–3), and high-risk (3.5–18 points).

This study was approved by the Institutional Review Board (IRB) of Kyung Hee University (IRB No. KHGIRB-24-307; Approval Date: June 24, 2024). The study’s ethical guidelines were based on the Declaration of Helsinki. All participants provided informed consent prior to participation.

### 2.2 Sociodemographic characteristics

Sociodemographic data included information on gender, age (in years), education level (elementary school or lower, middle school, high school, university or higher), duration of living alone (<1 year, 1–3 years, 3–5 years, 5–10 years, ≥10 years), current employment status, average monthly household income (≥1,000,000 KRW, <1,000,000 KRW, equivalent to approximately ≥$750, <$750 USD), and average monthly food expenditure (KRW, with USD equivalents).

### 2.3 Health condition

Health status was assessed using BMI, number of chronic diseases (EA), and self-rated health status (very healthy, relatively healthy, average, unhealthy, and very unhealthy). Self-perceived age (in years) and interest in health (not very high, not high, high, or very high) were also assessed. Depression levels were measured using the 15-item Geriatric Depression Scale [GDS-15; (23)]. Responses are binary (yes/no) and scores are categorized as normal (0–5), mild depression (6–10), and severe depression (11–15). Loneliness was assessed using the 3-item UCLA Loneliness Scale [UCLA-3; (24)]. Items were rated as 1 (hardly ever), 2 (sometimes), or 3 (often). Scores of 3–5 indicated no loneliness, whereas scores of 6–9 indicated loneliness. Life satisfaction was measured using the Korean version of the Satisfaction with Life Scale (K-SWLS), rated on a 7-point scale (1 = strongly disagree, 7 = strongly agree), with higher scores indicating greater life satisfaction (25). Quality of life was evaluated using the EQ-5D-5L. The scale generates a single score ranging from −0.59 to 1, where 1 represents excellent health and 0 represents a state equivalent to death (26).

### 2.4 Oral health status

Oral health status was assessed using the Oral Health Impact Profile 14 [OHIP-14; (27)], a 14-item questionnaire rated on a 4-point scale, with higher scores indicating an increased severity of oral health issues. Swallowing difficulties were assessed using the Eating Assessment Tool [EAT-10; (28)], a 10-item questionnaire rated on a 4-point scale. Participants scoring ≥3 points were classified as being at risk for dysphagia. Chewing difficulty was measured using a 5-point Likert scale (1 = very difficult, 5 = not difficult at all). In this study, scores of 1–3 represented the experience of chewing difficulty, whereas scores of 4–5 represented no difficulty (29). Additional oral health indicators included tooth brushing frequency (twice or more per day), presence of periodontitis, gum swelling, and dry mouth severity. Dry mouth was assessed using a Numeric Rating Scale (NRS; 0–10 points), and the number of missing teeth was also recorded.

### 2.5 Nutritional and dietary assessment

Nutritional status was evaluated using the Mini Nutritional Assessment (MNA), a tool designed for comprehensive nutritional assessment in older adults (30). Based on their scores, participants were classified into three groups: normal nutrition, at-risk of malnutrition, and malnourished.

### 2.6 Statistical analyses

Statistical analyses were performed using SPSS Statistics (version 28). To analyze group differences in oral frailty, the Oral Frailty Screening tool was used as the dependent variable. For sociodemographic characteristics, chi-square tests and one-way ANOVA were used. After adjusting for age and gender as covariates, ANCOVA was used for continuous variables and Generalized Linear Models (GLM) were applied to categorical variables to analyze differences in health status as well as oral and nutritional status. Finally, ordinal logistic regression was performed to identify factors influencing nutritional status and oral frailty among older adults living alone. Dependent variables were nutritional status (1 = normal, 2 = at risk of malnutrition, 3 = malnutrition) and oral frailty (1 = normal, 2 = at risk, 3 = high risk). The results were presented as odds ratios (OR) with 95% confidence intervals (CI), and statistical significance was determined at *p* < 0.05.

## 3 Results

### 3.1 Sociodemographic factors

A comparison of sociodemographic factors based on oral frailty status is presented in Table 1. Participants had an average age of 80.1 years and approximately 90% of them were female. Additionally, 76.4% had completed only elementary school or lower and a majority (64.4%) had been living alone for 10 years or more. In terms of income, 92.7% of participants had a monthly income of less than 1,000,000 KRW. Food expenditure varied, with 48.0% participants spending over 300,000 KRW per month and 40.3% spending between 160,000 and 300,000 KRW. Regarding area of residence, 66.5% of participants lived in rural areas, and no significant difference in oral frailty status was found across urban and rural groups (*p* = 0.219). Of all the sociodemographic factors, only food expenditure showed a significant difference based on oral frailty status (*p* = 0.049), with the high-risk oral frailty group having the highest proportion of participants spending 150,000 KRW or less on food.

**Table 1:** Socioeconomic factors according to oral function.

Variable	Total (*N* = 606)	Normal (*N* = 121)	At risk (*N* = 213)	High risk (*N* = 272)	*p*-value
Gender					0.807
Women	541 (89.3)	110 (90.9)	189 (88.7)	242 (89.0)	
Man	65 (10.7)	11 (9.1)	24 (11.3)	30 (11.0)	
Age (yrs)	80.1 ± 5.8	78.6 ± 5.2	80.3 ± 6.0	80.6 ± 5.9	0.015
Area					0.219
Urban	203 (33.5)	38 (31.4)	81 (38.0)	84 (30.9)	
Rural	403 (66.5)	83 (68.6)	132 (62.0)	188 (69.1)	
Educational level					0.671
≤Elementary	463 (76.4)	89 (73.6)	168 (78.9)	206 (75.7)	
Middle, High	135 (22.3)	31 (25.6)	43 (20.2)	61 (22.4)	
≥College	8 (1.3)	1 (0.8)	2 (0.9)	5 (1.8)	
Duration of living alone					0.320
Less than 1 year	35 (5.8)	9 (7.4)	8 (3.8)	18 (6.6)	
1 to 5 years	77 (12.7)	14 (11.6)	28 (13.1)	35 (12.9)	
5 to 10 years	104 (17.2)	27 (22.3)	30 (14.1)	47 (17.3)	
≥10 years	390 (64.4)	71 (58.7)	147 (69.0)	172 (63.2)	
Employment status					0.197
Currently	188 (31.0)	45 (37.2)	59 (27.7)	84 (30.9)	
None	418 (69.0)	76 (62.8)	154 (72.3)	188 (69.1)	
Household income (month, KRW)					0.124
≤1,000,000	562 (92.7)	107 (88.4)	200 (93.9)	255 (93.8)	
>1,000,000	44 (7.3)	14 (11.6)	13 (6.1)	17 (6.3)	
Food purchase cost (month, KRW)					0.049
≤ 150,000	71 (11.7)	9 (7.4)	22 (10.3)	40 (14.7)	
160,000 ~ 300,000	244 (40.3)	48 (39.7)	78 (36.6)	118 (41.9)	
>300,000	291 (48.0)	64 (52.9)	113 (53.1)	114 (41.9)	

Data was presented as mean ± standard deviation (SD) for continuous variables and frequency (percentage) for categorical variables. *p*-value was calculated by one-way analysis of variance (ANOVA) for continuous variables and chi-square test for categorical variables.

### 3.2 Health condition and lifestyle factors

Table 2 presents an analysis of participants’ health status, lifestyle factors, and quality of life, adjusted for age and gender. Participants had an average of 2.86 chronic diseases, with significantly more chronic diseases in the at-risk and high-risk oral frailty groups compared to the normal group (*p* < 0.001). Self-rated health status also declined with increasing oral frailty: 20.7% of the normal group reported their health as poor or very poor compared to 44.2% in the at-risk group and 50.0% in the high-risk group (*p* < 0.001). On average, participants perceived their health age as 77.7 years, 2.41 years younger than their actual age. The difference between chronological age and subjective age also varied significantly by oral frailty status, with the normal group reporting the largest gap (3.00 years) and the high-risk group the smallest (1.97 years) (*p* = 0.037). Additionally, 75.7% of participants reported having little to no interest in their health. The high-risk oral frailty group had the highest proportion of older adults (19.5%; *p* < 0.001) experiencing severe depression. Similarly, UCLA-3 loneliness scores were significantly higher in the at-risk and high-risk oral frailty groups compared to those in the normal group (*p* = 0.017). Life satisfaction declined with increasing oral frailty, with significant differences between scores of at-risk and normal groups (*p* = 0.028), and quality of life scores followed a similar trend (*p* < 0.001).

**Table 2:** Health condition and life-related factors according to oral function.

Variable	Total (*N* = 606)	Normal (*N* = 121)	At risk (*N* = 213)	High risk (*N* = 272)	*p*-value
Body Mass Index (BMI)					0.386
Underweight (<18.5)	45 (7.4)	3 (2.5)	17 (8.0)	25 (9.2)	
Normal weight (18.5–24.9)	426 (70.3)	93 (76.9)	149 (70.0)	184 (67.6)	
Overweight (≥25)	132 (22.3)	25 (20.7)	47 (22.1)	63 (23.2)	
Number of disease	2.86 ± 1.67	2.2 ± 1.46	2.9 ± 1.56	3.1 ± 1.76	< 0.001
Subjective health recognition					<0.001
Very bad	39 (6.4)	4 (3.3)	11 (5.2)	24 (8.8)	
Bad	216 (35.6)	21 (17.4)	83 (39.0)	112 (41.2)	
Moderate	250 (41.3)	66 (54.5)	82 (38.5)	102 (37.5)	
Good	93 (15.4)	28 (23.1)	34 (16.0)	31 (11.4)	
Very good	8 (1.3)	2 (1.7)	3 (1.4)	3 (1.1)	
Subjective Age (yrs)	77.7 ± 7.46	75.74 ± 6.59	77.67 ± 8.33	78.59 ± 6.96	0.037
(Age) - (Subject age)	2.41 ± 6.73	3.00 ± 5.29	2.64 ± 7.63	1.97 ± 6.53	0.037
Health interest					0.269
Almost never	109 (18.0)	22 (18.2)	38 (17.8)	49 (18.0)	
Not much	350 (57.7)	79 (65.3)	122 (57.3)	149 (54.8)	
Somewhat	140 (23.1)	20 (16.5)	50 (23.5)	70 (25.7)	
Very High	7 (1.2)	0 (0.0)	3 (1.4)	4 (1.5)	
Depression (GDS-15)					<0.001
Normal	331 (54.6)	91 (75.2)	116 (54.5)	124 (45.6)	
Moderate depression	196 (32.3)	25 (20.7)	76 (35.7)	95 (34.9)	
Severe depression	79 (13.0)	5 (4.1)	21 (9.9)	53 (19.5)	
Isolation (UCLA-3)					0.017
Not lonely	224 (37.0)	57 (47.1)	65 (30.5)	102 (37.5)	
Lonely	382 (63.0)	64 (52.9)	148 (69.5)	170 (62.5)	
Life satisfaction (K-SWLS)	14.32 ± 4.59	15.25 ± 4.71	14.23 ± 5.04	13.99 ± 4.12	0.028
Quality of life (EQ-5D)	0.75 ± 0.15	0.82 ± 0.14	0.76 ± 0.14	0.72 ± 0.15	< 0.001

Data are presented as mean ± standard deviation (SD) for continuous variables and frequency (percentage) for categorical variables. *p*-values were adjusted for age and gender using analysis of covariance (ANCOVA) for continuous variables and generalized linear models (GLiM) for categorical variables.

### 3.3 Oral health and nutritional status

Analyses of oral health status and nutritional assessment based on oral frailty status, adjusted for age and gender, are presented in Table 3. The average OHIP-14 score was 17.55, with significantly higher scores in the at-risk and high-risk oral frailty groups (*p* < 0.001). A similar pattern was observed for the EAT-10 scores, indicating a higher risk of dysphagia in the at-risk (19.7%) and high-risk (25.4%) groups than in the normal group (0.8%; *p* < 0.001). Chewing difficulty rates differed significantly by oral frailty status, with an overall rate of 44.2% and the highest rate being observed in the high-risk oral frailty group (53.3%). Participants with better oral frailty status reported less difficulty in chewing (*p* < 0.001). The frequency of tooth brushing at least twice a day was significantly lower in the high-risk group (*p* = 0.013). The prevalence of periodontitis increased with the severity of oral frailty (*p* = 0.006), with an average prevalence of 62.9%. Additionally, significant differences were found between the groups in terms of gingival swelling, and number of missing teeth. Participants in the high-risk oral frailty group reported the highest levels of dry mouth, with a mean score of 3.75 on the Numeric Rating Scale (*p* < 0.001). In contrast, the salivary flow rate, as measured by the Salivary Flow Rate Test, decreased significantly with increasing oral frailty severity (*p* = 0.005). The normal group showed the highest average secretion (3.88 cm), while the high-risk group had the lowest (3.34 cm), suggesting a clear decline in salivary function associated with worsening oral frailty. Regarding nutritional status, 51.0% of participants were at risk for malnutrition and 9.6% were classified as malnourished. Malnutrition prevalence was significantly higher in the high-risk oral frailty group (*p* = 0.004).

**Table 3:** Oral health and nutrition status according to oral function.

Variable	Total (*N* = 606)	Normal (*N* = 121)	At risk (*N* = 213)	High risk (*N* = 272)	*p-*value
OHIP-14	17.55 ± 11.57	12.22 ± 9.04	17.34 ± 11.61	20.08 ± 11.75	< 0.001
EAT-10	1.74 ± 3.74	0.17 ± 0.86	1.97 ± 4.30	2.25 ± 3.89	< 0.001
Dysphagia (EAT-10)					< 0.001
At risk	112 (18.5)	1 (0.8)	42 (19.7)	69 (25.4)	
No risk	494 (81.5)	120 (99.2)	171 (80.3)	203 (74.6)	
Chewing difficulty					< 0.001
Yes	268 (44.2)	28 (23.1)	95 (44.6)	145 (53.3)	
No	338 (55.8)	93 (76.9)	118 (55.4)	127 (46.7)	
Brush teeth (more than twice a day)					0.013
No	29 (4.8)	1 (0.8)	9 (4.2)	19 (7.0)	
Yes	577 (95.2)	120 (99.2)	204 (95.8)	253 (93.0)	
Periodontitis					0.006
Yes	381 (62.9)	62 (51.2)	136 (63.8)	183 (67.3)	
No	225 (37.1)	59 (48.8)	77 (36.2)	89 (32.7)	
Gingival swelling					<0.001
Yes	288 (47.5)	37 (30.6)	99 (46.5)	152 (55.9)	
No	318 (52.5)	84 (69.4)	114 (53.5)	120 (44.1)	
Numeric rating scale	3.51 ± 2.43	2.70 ± 1.93	3.66 ± 2.41	3.75 ± 2.58	< 0.001
Salivary flow rate test (cm)	3.55 ± 1.52	3.88 ± 1.49	3.62 ± 1.42	3.34 ± 1.59	0.005
Tooth loss (number)	9.29 ± 9.71	6.82 ± 8.52	8.42 ± 9.51	11.06 ± 10.06	< 0.001
Nutritional status (MNA)					0.004
Adequate	239 (39.4)	64 (52.9)	84 (39.4)	91 (33.5)	
At risk of malnutrition	309 (51.0)	51 (42.1)	113 (53.1)	145 (53.3)	
Malnourished	58 (9.6)	6 (5.0)	16 (7.5)	36 (13.2)	

Data are presented as mean ± standard deviation (SD) for continuous variables and frequency (percentage) for categorical variables. *p*-values were adjusted for age and gender using analysis of covariance (ANCOVA) for continuous variables and generalized linear models (GLiM) for categorical variables.

### 3.4 Factors affecting nutritional status

Logistic regression analysis examined factors affecting the nutritional status of older adults living alone (Table 4). Poorer self-rated health status was associated with a 68% higher risk of malnutrition (OR = 1.68, 95% CI: 1.30–2.17, *p* = 0.000). Higher scores on the UCLA-3 loneliness scale were also significantly associated with an increased risk of malnutrition (OR = 1.09, 95% CI: 1.00–1.20, *p* = 0.048). Further, poorer oral health-related quality of life (higher OHIP-14 scores; OR = 1.02, 95% CI: 1.01–1.04, *p* = 0.007) and greater swallowing difficulties (higher EAT-10 scores; OR = 1.08, 95% CI: 1.02–1.14, *p* = 0.004) were associated with an increased risk of malnutrition. Higher oral frailty scores (OR = 1.10, 95% CI: 1.02–1.18, *p* = 0.008) and not brushing teeth at least twice daily (OR = 4.28, 95% CI: 1.90–9.62, *p* = 0.000) significantly increased the risk of malnutrition. Conversely, a higher level of health interest (OR = 0.77, 95% CI: 0.60–0.99, *p* = 0.039) and greater life satisfaction (K-SWLS scores; OR = 0.95, 95% CI: 0.91–0.99, *p* = 0.009) were associated with a lower risk of malnutrition.

**Table 4:** Factors affecting nutritional status.

Variable	OR (95% CI)	*p-*value
Gender		
Women	1.46 (0.84–2.54)	0.178
Man	Ref	-
Age (yrs)	1.03 (1–1.06)	0.091
Employment status		
Currently	0.72 (0.49–1.04)	0.082
None	Ref	-
Duration of living alone	0.9 (0.75–1.08)	0.257
Number of disease	0.92 (0.82–1.02)	0.122
Subjective health status	1.68 (1.3–2.17)	0.000
Health interest	0.77 (0.6–0.99)	0.039
Depression (GDS-15, Score)	0.96 (0.91–1.02)	0.162
Isolation (UCLA-3, Score)	1.09 (1–1.2)	0.048
Life satisfaction (K-SWLS, Score)	0.95 (0.91–0.99)	0.009
Quality of life (EQ-5D, Score)	0.28 (0.07–1.14)	0.076
OHIP-14 (Score)	1.02 (1.01–1.04)	0.007
Dysphagia (EAT-10, Score)	1.08 (1.02–1.14)	0.004
Oral frailty (Score)	1.1 (1.02–1.18)	0.008
Brush teeth (more than twice a day)		
No	4.28 (1.9–9.62)	0.000
Yes	Ref	-
NRS numeric rating scale	1.05 (0.97–1.13)	0.194

OR, Odds Ratio; CI, 95% Confidence Interval.

Dependent variable = MNA (1 = Adequate, 2 = At risk of malnutrition, 3 = Malnourished).

### 3.5 Factors affecting oral frailty

Logistic regression analysis of the factors affecting oral frailty are presented in Table 5. An increase in the number of chronic diseases by one, increased the likelihood of worsening oral frailty by approximately 21% (OR = 1.21, 95% CI: 1.09–1.34, *p* = 0.000). Increasing social isolation (UCLA-3) scores were associated with increased risk of oral frailty (OR = 1.09, 95% CI: 1.00–1.20, *p* = 0.021). Tooth loss increased the odds of worsening oral frailty by approximately 2% per lost tooth (OR = 1.02, 95% CI: 1.01–1.04, *p* = 0.028). The presence of gingival edema was associated with 32% higher odds of worsening oral frailty (OR = 1.68, 95% CI: 0.48–0.97, *p* = 0.031). Compared to those with severe depression, participants with mild depression showed 54% lower odds of worsening oral frailty (OR = 0.46, 95% CI: 0.26–0.81, *p* = 0.008). Conversely, lower food purchase costs (OR = 0.78, 95% CI: 0.61–1.00, *p* = 0.046), higher quality of life scores (OR = 0.29, 95% CI: 0.08–1.00, *p* = 0.050), higher saliva secretion (OR = 0.85, 95% CI: 0.76–0.94, *p* = 0.002), and higher nutritional status scores (OR = 0.95, 95% CI: 0.90–1.00, *p* = 0.032) were all associated with a lower risk of oral frailty.

**Table 5:** Factors affecting oral frailty.

Variable	OR (95% CI)	*p-*value
Gender		
Women	0.59 (0.34–1.02)	0.061
Man	Ref	-
Age (yrs)	1.03 (1–1.06)	0.095
Educational level	1.32 (0.92–1.9)	0.136
Employment status		
Currently	1.27 (0.89–1.82)	0.183
None	Ref	-
Household income	1 (0.99–1)	0.123
Food purchase cost	0.78 (0.61–1)	0.046
Number of disease	1.21 (1.09–1.34)	0.000
Depression (GDS-15)		
Normal	0.29 (0.16–0.5)	0.000
Moderate depression	0.46 (0.26–0.81)	0.008
Severe depression	Ref	-
Isolation (UCLA-3, Score)	0.91 (0.83–0.99)	0.021
Quality of Life (EQ-5D, Score)	0.29 (0.08–1)	0.050
OHIP-14 (Score)	1.02 (1–1.03)	0.066
Brush teeth (more than twice a day)		
No	1.55 (0.68–3.57)	0.299
Yes	Ref	-
Salivary flow rate test (cm)	0.85 (0.76–0.94)	0.002
Tooth loss (number)	1.02 (1–1.04)	0.028
Gingival swelling		
Yes	0.68 (0.48–0.97)	0.031
No	Ref	-
Salivary flow rate test (cm)	0.85 (0.76–0.94)	0.002
Nutritional status (MNA, Score)	0.95 (0.9–1)	0.032

OR, Odds Ratio; CI, 95% Confidence Interval.

Dependent variable = Oral Frailty (1 = Good, 2 = At risk, 3 = High risk).

## 4 Discussion

This study aimed to assess oral frailty among older adults living alone and analyze the impact of their oral function on nutritional status. The average age of participants was 80.1 years, with 44.9% of them classified as high-risk for oral frailty. The findings indicate that individuals at higher risk tended to have lower food expenditure—a significant factor influencing oral frailty. Among South Korean households with older adults, food expenses constitute the largest share of total expenditure, with those living alone facing greater economic burden (31). The average monthly income of older adults living alone in South Korea is approximately 1.57 million KRW and participants in this study had incomes below 1 million KRW, suggesting that economic status and food expenditure are strongly associated with oral frailty (32). Previous research suggests that lower economic status is associated with increased consumption of energy-dense diets and refined carbohydrates, as well as limited intake of nutrient-dense foods such as meat and dairy products. This dietary imbalance can contribute to oral frailty, particularly as they lead to deficiencies in essential nutrients such as calcium, vitamin C, and vitamin E, which are crucial for oral health (33–35). Although providing financial support alone may not fully address these challenges, implementing targeted meal support programs combined with preventive measures could help mitigate oral health deterioration and maintain overall health.

According to the 2023 Elderly Survey in South Korea, the average number of chronic diseases per older adult was 2.2, whereas in this study, the average for individuals in the high-risk oral frailty group was 3.1, suggesting a greater burden of illness (36). These findings are consistent with extant literature that links a higher risk of oral frailty to chronic conditions such as diabetes, pneumonia, and heart disease (37). Additionally, individuals with severe oral frailty reported poorer self-rated health and a chewing difficulty rate of 44.2%—significantly higher than the national average of 30.2% as reported in the South Korea Community Health Survey (38). Only 27.5% participants had more than 20 remaining teeth, a number considerably lower than the 52.3% reported in the Korea National Health and Nutrition Examination Survey, highlighting a more severe oral health crisis among vulnerable older adults (39).

Oral frailty and nutritional status demonstrated a direct association. Poor oral health conditions, including pain, infection, and tooth loss, can substantially impact food intake. Conversely, inadequate nutrient intake can adversely affect teeth, periodontal tissues, and salivary glands, aggravating oral discomfort. Systemic diseases associated with malnutrition often present with oral symptoms, exacerbating the risk of oral frailty (18, 40). These long-term, cyclical conditions contribute to increased frailty and mortality, emphasizing the need for early screening, targeted interventions, and consistent oral hygiene care among older adults (41). Maintaining natural teeth and regular tooth brushing are critical factors in reducing frailty risk (42), with adequate hydration being essential for preventing dry mouth and sustaining oral health (43).

Loneliness emerged as a key common factor affecting both nutritional status and oral frailty. Studies have found that older adults living alone experience higher levels of loneliness and depression than those living in multi-person households (44). Additionally, tooth loss and oral pain are associated with increased risk of depression, which in turn reduces social participation and deepening feelings of isolation (45–47). Loneliness has also been identified as a predictor of malnutrition risk in older adults (48). These findings highlight the need for comprehensive care strategies that integrate emotional support, human-centered care, and regular oral hygiene education to mitigate depression and loneliness while also improving self-rated health and self-esteem. Strategies such as home visits, counseling, and routine health monitoring should be implemented to encourage healthier dietary choices and eating habits. Preemptive and continuous health checkups and interventions are essential to maintain the well-being of older adults living alone.

This study has certain limitations as its findings cannot be generalized to all South Korean older adults who live alone. Additionally, the oral frailty assessment tool we used was a screening instrument rather than a diagnostic tool. Although urban and rural classification was considered, more detailed aspects of healthcare accessibility such as distance to services or ease of utilization were not fully captured in this study. These contextual factors may influence older adults’ ability to maintain oral health and thus warrant further exploration. However, this study’s strengths include its large sample size and comprehensive analysis using validated tools to assess oral health, nutrition, psychological well-being, and quality of life. These factors distinguish it from previous studies. Given the rapid aging of South Korea’s population and the increasing proportion of older adults living alone, this study underscores the urgent need for oral and nutritional care in community health management, especially for older adults who may otherwise be neglected. Future studies could benefit from incorporating oral health screening into home-based nutrition services and exploring linkage strategies with local dental providers to support those at risk. Further research is essential to develop strategies that promote healthy aging and improve older adult’s quality of life.

## Data Availability

The data are not publicly available due to the potential risk of participant re-identification. However, de-identified data can be made available upon reasonable request to the corresponding author.
